# Poly(3-hexylthiophene)-Based Organic Thin-Film Transistors with Virgin Graphene Oxide as an Interfacial Layer

**DOI:** 10.3390/polym14235061

**Published:** 2022-11-22

**Authors:** Eyob N. Tarekegn, Mastooreh Seyedi, Igor Luzinov, William R. Harrell

**Affiliations:** 1 Holcombe Department of Electrical and Computer Engineering, Clemson University, Clemson, SC 29634, USA; 2 Department of Materials Science and Engineering, Clemson University, Clemson, SC 29634, USA

**Keywords:** OTFT, P3HT, graphene oxide, graphene oxide interfaces, polymer semiconductor layers, improvement of P3HT interface

## Abstract

We fabricated and characterized poly(3-hexylthiophene-2, 5-diyl) (P3HT)-based Organic thin-film transistors (OTFTs) containing an interfacial layer made from virgin Graphene Oxide (GO). Previously chemically modified GO and reduced GO (RGO) were used to modify OTFT interfaces. However, to our knowledge, there are no published reports where virgin GO was employed for this purpose. For the sake of comparison, OTFTs without modification were also manufactured. The structure of the devices was based on the Bottom Gate Bottom Contact (BGBC) OTFT. We show that the presence of the GO monolayer on the surface of the OTFT’s SiO_2_ dielectric and Au electrode surface noticeably improves their performance. Namely, the drain current and the field-effect mobility of OTFTs are considerably increased by modifying the interfaces with the virgin GO deposition. It is suggested that the observed enhancement is connected to a decrease in the contact resistance of GO-covered Au electrodes and the particular structure of the P3HT layer on the dielectric surface. Namely, we found a specific morphology of the organic semiconductor P3HT layer, where larger interconnecting polymer grains are formed on the surface of the GO-modified SiO_2_. It is proposed that this specific morphology is formed due to the increased mobility of the P3HT segments near the solid boundary, which was confirmed via Differential Scanning Calorimetry measurements.

## 1. Introduction

Organic thin-film transistors (OTFTs) are attractive because of their substantial potential in applications where low cost, large surface area, and flexible structures are required. Some of the most widely used applications of OTFTs are flat panel displays [1], sensors [2], radio frequency identification (RFID) tags [3], and medical devices [4]. OTFTs have come a long way since they were first reported in 1986 [5], and particularly in the past two decades, there has been significant progress in the fabrication process and performance of OTFTs. However, their performance is still not on par with their inorganic counterparts. In particular, the field-effect mobility and switching speed of OTFTs are lower, and the interfacial stability at the dielectric/semiconductor interface and electrode/semiconductor interface are much less than in Si-based devices, especially with regard to trapped charge and interface states. 

OTFTs turn on by applying a gate voltage of appropriate polarity and magnitude, accumulating majority charge carriers at the dielectric/organic semiconductor interface and, therefore, forming a conducting channel between the source and the drain. Charge carriers are injected from the source electrode into the organic semiconductor (or channel) and extracted from the semiconductor into the drain electrode by applying a voltage at the drain terminal with respect to the source. The operation of these transistors is significantly affected by the interfaces present in the device, and properly engineered interfaces can significantly improve the device’s performance [6,7,8,9,10]. Two of the most important interfaces that determine the performance of bottom gate bottom contact (BGBC) OTFTs, explored in this work, are the electrode/organic semiconductor and dielectric/organic semiconductor interfaces [8] 

The electrode/organic semiconductor interface is responsible for injecting charge carriers into the organic semiconductor, which functions as the transistor channel. The dielectric/organic semiconductor interface is responsible for the charge carrier transport in the channel. Once the charge carriers are injected into the organic semiconductor, they are transported across the channel near the dielectric/organic semiconductor interface. Since most of this transport occurs within a few molecular layers of the organic semiconductor near the dielectric surface [9], the dielectric surface can significantly impact the charge carrier transport. During the deposition of the organic semiconductor, the surface roughness, the surface energy, and functionality of the dielectric profoundly influences the structure of the organic semiconductor layer, such as the molecular ordering, molecular orientation, and morphology of the organic semiconductor [9,11,12,13].

The OTFT’s interfaces are typically engineered via the deposition of nanoscale layers between the electrode/dielectric and the organic semiconductor layer. To this end, significant knowledge is accumulated on effective modification of the interfaces with self-assembled monolayers (SAMs) [9,14,15,16,17,18,19,20], transition metal oxides [21], and tetracyanoquinodimethane (TCNQ) [22,23]. Recently, graphene oxide (GO) has been explored to modify the devices’ electrode and dielectric surfaces to improve their performance [24,25,26]. GO was chosen for the interfacial modification because graphene-based materials, including GO, are chemically compatible with organic semiconductors and have already been explored in the fabrication of a number of devices [24,27,28,29,30,31]. Thus, it is a suitable surface for depositing organic semiconductors. GO is also compatible with Au electrodes. GO surfaces possess a high surface energy [24], which shows good wetting properties with polar and non-polar solvents. Furthermore, GO is relatively inexpensive, has low toxicity, and is compatible with photolithographic fabrication processes. It has also been shown that using a covalently linked GO-Au electrode improves the charge injection process of OTFTs [24]. 

To date, scientific publications have reported the employment of either chemically modified/anchored GO or reduced GO (RGO) for the modification of BGBC OTFT interfaces. To the best of our knowledge, there are no published reports where virgin GO was employed for this purpose. To this end, we investigated the impact of a virgin (“as-received”) GO localization at the electrode/organic semiconductor interface and the dielectric/organic semiconductor interface. Specifically, we determined that the drain current and field-effect mobility of the OTFTs were significantly increased by modifying the interfaces with GO nanoscale layers. We associate the obtained enhancement with the previously reported [24] decrease in contact resistance for GO-covered electrodes and the particular structure of the poly(3-hexylthiophene-2, 5-diyl), P3HT, polymeric semiconductor layer on the dielectric surface.

## 2. Experimental Section

### 2.1. Fabrication

We fabricated poly(3-hexylthiophene-2, 5-diyl) (P3HT)-based OTFTs without a GO interfacial layer using a photolithographic fabrication process. These devices hereinafter are referred to as standard OTFTs, denoted as S-OTFTs. Second, we fabricated P3HT-based OTFTs containing a GO interfacial layer using the same photolithographic fabrication process. The devices with the GO interfacial layer, hereinafter referred to as modified OTFTs, are denoted as M-OTFTs. The structure of both devices was based on the BGBC OTFT structure. The details of the fabrication process are reported in our previous publication [32].

**Figure 1:**
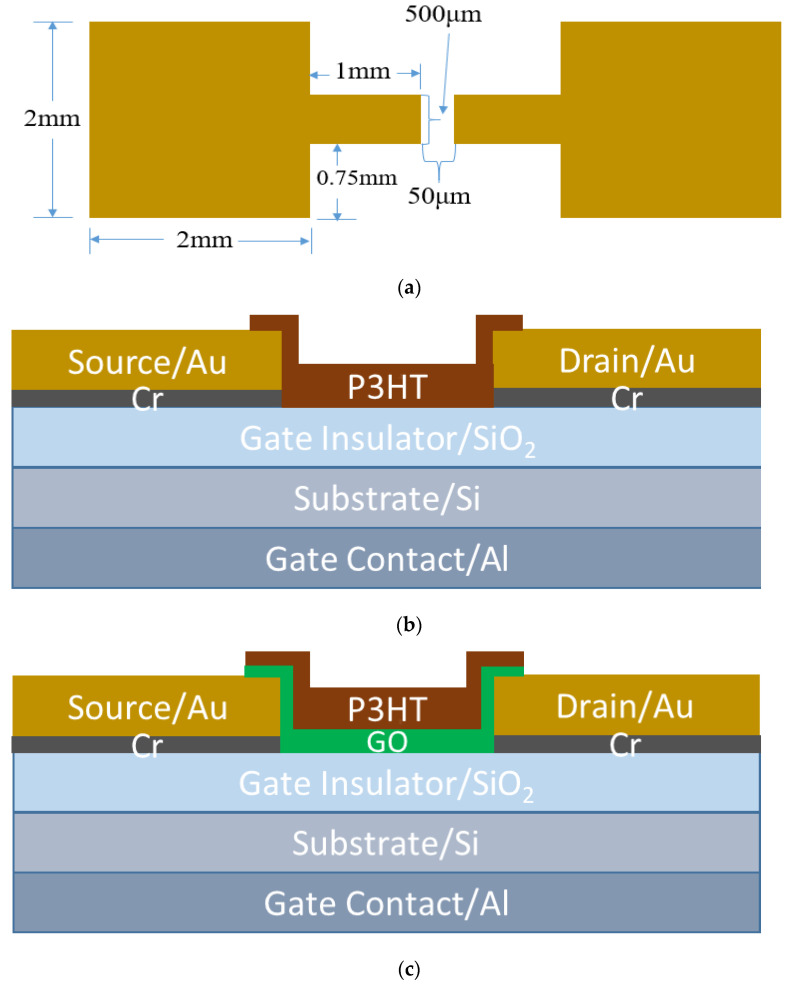
(**a**) Top view of the source and drain contacts with their dimensions; (**b**) Schematic structure of P3HT-based S-OTFT; (**c**) Schematic structure of P3HT-based M-OTFT.

The S-OTFTs were fabricated on thermally oxidized silicon wafers obtained from UniversityWafer, Inc., with a diameter of 100 mm. The substrate was a 500 µm thick degenerately doped p-type silicon wafer with a resistivity in the range of 0.001–0.005 Ω-cm. The degenerate doping was chosen in order to form a good low-resistance ohmic contact with the gate metal. The gate insulator was a 300 nm thick SiO_2_ layer that was thermally grown on the silicon substrate. The gate contact was made from aluminum and was deposited onto the wafer backside using an E-beam evaporator (CHA Mark 40, CHA Industries, Inc., Fremont, CA, USA). The source and drain contacts were made of gold and deposited onto the wafer’s front side with a thin adhesion layer of chromium using the E-beam evaporator. Source and drain contacts were patterned using a photolithography and lift-off process. The dimensions of the conductive channel length and width of the device were 50 µm and 500 µm, respectively. The top view of the source and drain contacts is illustrated in Figure 1a. 

P3HT, a regioregular electronic grade with a regioregularity greater than or equal to 90%, was used as an organic semiconductor. The P3HT was obtained from Rieke Metals in powder form. According to the manufacturer, the polymer has a molecular weight of 50,000–70,000 g/mole. Chloroform was used to obtain a 0.48 wt % P3HT solution. The P3HT was spin-coated onto the wafer at an acceleration of 1000 rpm/s until a maximum speed of 2000 rpm was reached and maintained for 60 s, and subsequently annealed at 110 °C for 1 h. The thickness of the resulting P3HT layer was measured using a profilometer and determined to be 58 nm. The P3HT was then patterned using a photolithography process. 226 S-OTFTs were fabricated on a single wafer. The schematic structure of the device is illustrated in Figure 1b. As illustrated in the figure, the P3HT was not only limited to the channel area (between the edges of the drain and the source) but has a slight overlap onto the electrodes. The devices were designed in this manner to ensure complete coverage of the channel and the Source/Drain sidewalls and create a larger contact area between the electrode and the organic semiconductor [32]. Increasing the contact area results in lower contact resistance and a higher injection rate of charge carriers into the channel, which improves device performance [8,22].

The fabrication of M-OTFTs followed a nearly identical fabrication process, except for adding a GO layer underneath the P3HT. An aqueous dispersion of GO was obtained from Goographene, Inc. It was received with a concentration of 5 mg/mL in water. According to the manufacturer, the dimensions of the GO sheets were several hundred nanometers up to several micrometers in the XY plane and 0.7–1.2 nm in thickness [33]. The GO suspension was further diluted to 0.5 mg/mL with DI water. Once the gate, drain and source contacts were deposited, the GO solution was ready to be coated. The GO solution was agitated in an ultrasonic bath for 4 min and immediately spin-coated onto the wafer at an acceleration of 500 rpm/sec until a maximum speed of 1000 rpm was reached and maintained for 60 s, and subsequently baked at 110 °C for 40 min. The thickness of the GO was measured from an Atomic Force Microscopy (AFM) image and determined to be 1.5 nm. The P3HT was then spin-coated on top of the GO layer at an acceleration of 1000 rpm/sec until a maximum speed of 2000 rpm was reached and maintained for 60 s, and subsequently baked at 110 °C for 1 h. Both the GO and the P3HT layers were then patterned together in the same way as the standard devices. The dimensions of the conductive channel were essentially identical to the standard devices. 226 M-OTFTs were fabricated on a single wafer. The schematic structure of the modified device is illustrated in Figure 1c, and it is identical to the standard devices except for the GO interfacial layer. As illustrated in Figure 1c, the P3HT/GO was not only limited to the channel area but has an overlap onto the electrodes. As explained for the standard devices, these devices were designed this way to increase the contact area between Au and P3HT/GO [32].

### 2.2. Characterization

Current–voltage (I-V) measurements were performed using an HP-4156B Semiconductor Parameter Analyzer. The drain current vs. drain voltage (I_DS_-V_DS_) measurements were performed by applying a drain voltage from 0 V to −70 V with an increment of −0.5 V while biasing the gate with voltages ranging from 0 V to −60 V with an increment of −20 V. The drain current vs. gate voltage (I_DS_-V_GS_) measurements were performed by applying a gate voltage from 20 V to −60 V while biasing the drain at −5 V.

Atomic force microscopy (AFM, Dimension 3100, Veeco Digital Instruments, Inc., Santa Barbara, CA, USA) was used in tapping mode to investigate the morphology of the GO and P3HT layers. A Tencore Alpha Step 200 Profilometer was used to measure the thickness of the P3HT. Contact angle measurements of the immersion liquids (hexadecane and water) were performed using a KRUSS DSA 10 Drop Shape Analyzer at 20 s after droplet deposition on the GO and SiO_2_ surface. Differential scanning calorimetry (DSC) (Model 2920; TA Instruments, New Castle, DE, USA) was carried out at a heating/cooling rate of 20 °C/min and a temperature range of −100 °C to 150 °C. Model samples for DSC studies were prepared by addition of SiO_2_, GO, or SiO_2_-GO dispersion in chloroform to a P3HT chloroform solution, sonication of the mixture for 1 h at room temperature (RT), drying at RT, and annealing at 110 °C for 2 h. The obtained materials had the following composition in terms of the weight ratios: SiO_2_:P3HT/1:1, GO:P3HT/1:1, and SiO_2_:GO:P3HT/1:1:1. To prepare the materials, SiO_2_, GO, or SiO_2_-GO dispersions in water were dried and redispersed in chloroform using an ultrasound bath. Sonication was conducted for 1 h at room temperature. To prepare the materials, silicon oxide powder, particle diameter 0.5 microns, from Alfa Aesar was used. 

## 3. Results and Discussion

### 3.1. Electrical Properties

#### 3.1.1. Voltage-Current Characterization

**Figure 2:**
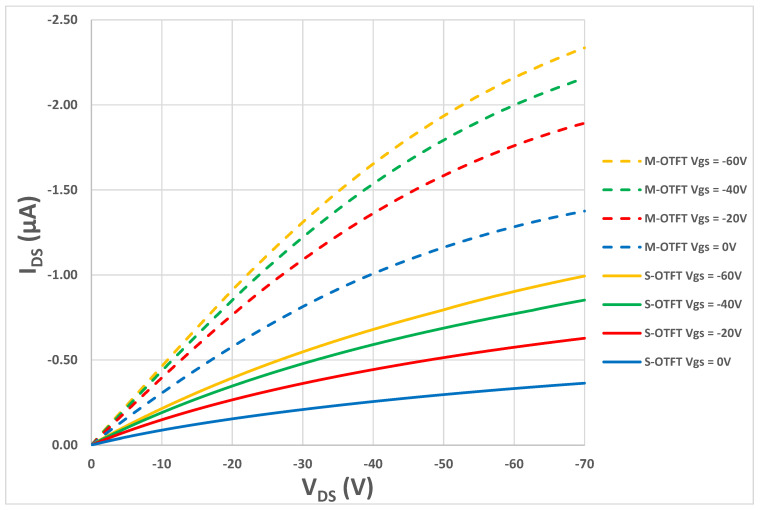
I_DS_-V_DS_ output characteristics of a S-OTFT vs. M-OTFT at different values of V_GS._ The solid lines are for a S-OTFT device and the broken lines are for a M-OTFT device.

Out of the 452 total OTFTs fabricated, more than 48 devices were randomly selected to perform electrical measurements. Half of the 48 devices were S-OTFTs, and the remaining half were M-OTFTs. In Figure 2, the output characteristics of two devices are shown, representing the majority of the measured devices. In this plot, the drain current is measured as a function of the drain voltage at various gate voltages. According to Figure 2, the drain current for the modified device is higher than for the standard device at all drain voltages and gate voltages. This shows that the channel for the modified device has a higher conductivity than the standard device. 

The threshold voltage and the mobility were extracted for all the transistors characterized. The threshold voltage is the minimum gate voltage required to accumulate charge carriers at the dielectric/organic semiconductor interface forming a conducting channel between the source and the drain [6]. The threshold voltage was extracted using an extrapolation in the linear region (ELR) method. This method consists of performing a linear extrapolation of the measured drain current vs. the gate voltage (I_DS_-V_GS_) curve to the V_GS_ axis with an applied drain voltage (*V_DS_*) that biases the device into the linear region [34]. The extrapolation is taken at the maximum slope of the I_DS_-V_GS_ curve. The slope of this curve, which is defined as the transconductance (*g_m_*), is given by:

(1) $$g_{m}=\frac{\partial I_{DS}}{\partial V_{GS}}$$

where I_DS_ is the drain to source current and V_GS_ is the gate to source voltage. The threshold voltage is then given by: [34,35]

(2) VTh=VGSi−VDS2

where *V_Th_* is the threshold voltage, *V_DS_* is the drain to source voltage, and *V_GSi_* is the V_GS_ axis intercept. 

**Figure 3:**
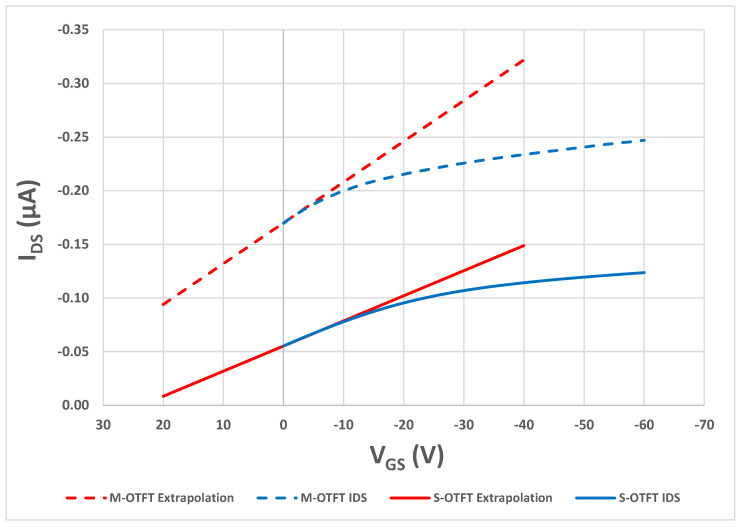
Transfer characteristics of a standard and modified OTFTs at V_DS_ = −5 V, along with the linear extrapolation of the curves at maximum transconductance. The solid lines are for a S-OTFT and the broken lines are for a M-OTFT.

The transfer curve of a S-OTFT at *V_DS_* = −5 V is shown with a solid blue line in Figure 3. The extrapolation line is shown in red. The threshold voltage is found to be 26.09 V. The transfer curve of a M-OTFT at *V_DS_* = −5 V is shown with a broken line in Figure 3, with the extrapolation line shown in red. The threshold voltage is found to be 47.24 V, which is significantly higher than the standard device. This high threshold voltage for the modified device is not optimal for low-power devices. 

#### 3.1.2. Mobility Determination

The mobilities of the devices were also extracted. Mobility is a measure of how fast charge carriers can move across the conducting channel per unit applied electric field. It determines the switching speed of a transistor. The relationship between the drain current, and the gate and drain voltages in an OTFT is described using the Compact DC model. In this model, the drain current can be expressed as [7,36,37]:

(3) $$I_{DS}=\frac{W}{L}C_{ox}µ\left[\left(V_{GS}-V_{Th}\right)V_{DS}-\frac{V_{DS}^{2}}{2}\right]$$

where *V_Th_* is the threshold voltage, V_DS_ is the drain to source voltage, W is the channel width, *L* is the channel length, µ is the field-effect mobility, and *C_ox_* is the dielectric or oxide capacitance per unit area. The oxide capacitance per unit area is given by,

(4) $$C_{OX}=\frac{\varepsilon_{i}\varepsilon_{o}}{t_{ox}}$$

where *ε_i_* is the dielectric constant of the gate insulator (3.9 for SiO_2_), *ε_o_* is the permittivity of free space, which has the value of 8.85×10−14 Fcm, and *t_ox_* is the gate insulator thickness.

The mobility of an OTFT can be calculated using Equation (3); however, there are two operating regions of the transistor: the linear and the saturation regions. For the linear region, *V_DS_* << (*V_GS_* − *V_Th_*); therefore, Equation (3) can be simplified in this case and written as:

(5) $$I_{DS}=\frac{W}{L}C_{OX}\mu_{lin}\left(V_{GS}-V_{Th}\right)V_{DS}$$

For the saturation region, *V_DS_* ≥ (*V_GS_ − V_Th_*). The onset of saturation occurs when V_DSsat_ = (*V_GS_ − V_Th_*). At the point of saturation, Equation (3) becomes,

(6) $$I_{DS}=\frac{W}{2L}C_{OX}\mu_{sat}{\left(V_{GS}-V_{Th}\right)}^{2}$$

To first order, *I_DS_* remains constant at its saturation value, I_DSat_, as *V_DS_* exceeds V_DSsat_. The terms $\mu_{lin}$ and $\mu_{sat}$ in Equations (5) and (6) refer to the field-effect mobility in the linear and saturation operation region, respectively. The field-effect mobility in the linear region, which is used in this work, can be extracted from Equation (5) and written as,

(7) $$\mu_{lin}=\frac{L}{WC_{OX}V_{DS}}g_{m}$$

where *g_m_* in Equation (7) is the maximum *g_m_*, which can be found from the transfer curves and can be calculated using Equation (1).

The field-effect mobility in the linear region of a typical standard device was found to be 4.21×10−3 cm2Vs, while for the modified device, it was found to be 7.3×10−3 cm2Vs. This result indicates that devices with a GO interfacial layer have higher mobility than devices with no interfacial layer. In general, the mobility values reported for the S-OTFT and M-OTFT here are higher than that reported for most BGBC P3HT-based devices in the scientific literature [38,39,40,41].

#### 3.1.3. GO Impact on Electrical Properties

The results reported above clearly show that the devices with the GO interfacial layer have a higher drain current and mobility than the standard devices. One of the reasons for this could be the electrical conductivity of the GO layer. GO is typically considered an electrical insulator, but its conductivity depends on the degree of reduction [42,43]. For example, reducing GO using thermal, chemical, electrochemical, or photochemical processes results in RGO, which is far more conductive than GO [42,43,44]. In this work, we used virgin GO “as-received” from the supplier without any reduction or chemical modification. However, to verify that the GO used here was not conducting and working in parallel with the P3HT, we performed a model experiment by fabricating devices with GO as the only active layer without any P3HT. The thickness of GO deposited was the same as that used to form an interfacial layer in the modified devices. I-V measurements were performed on these transistors, and no significant drain current was observed. Therefore, the enhanced drain current and field-effect mobility for the devices with the GO interfacial layer was not due to GO conductivity. This indicates that the observed enhanced transport properties are due to the modification of the source-drain electrodes and the dielectric surface in the channel area.

The modification of the source-drain electrodes with the GO layer can contribute to the enhancement of the drain current and the field-effect mobility of the modified devices. There must be certain compatibility between the electrode and the organic semiconductor interface in OTFTs; otherwise, the contact resistance at the interface and the molecular ordering of the organic semiconductor will reduce the injection efficiency of charge into the channel. It has been shown that Au is more compatible with GO than both p-type and n-type organic semiconductors [24]. An organic semiconductor deposited onto a GO-modified Au electrode will have less contact resistance and better molecular ordering than the one deposited on a pristine Au electrode [24]. Therefore, the devices with the GO interfacial layer have a higher injection of charges into the organic semiconductor because of the compatibility of GO with the Au source and drain electrodes, which ultimately contributes to the enhanced drain current and field-effect mobility.

### 3.2. Structural Characteristics

#### 3.2.1. Morphology via Atomic Force Microscopy

**Figure 4:**
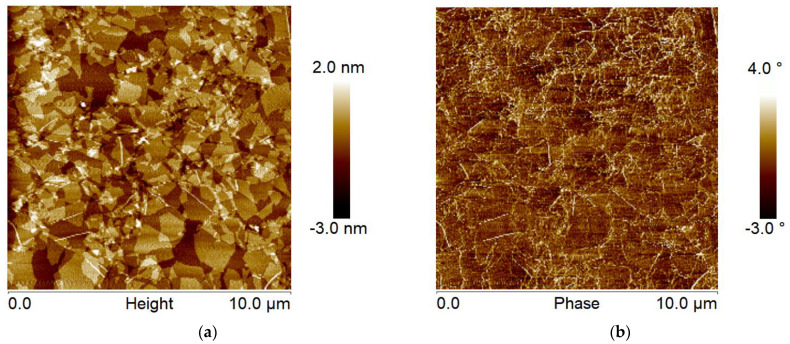
AFM topographical (**a**) and phase (**b**) images of a GO layer on a SiO_2_ surface.

The other factor contributing to the higher drain current and mobility of the modified OTFTs is the morphological and structural change of the P3HT when it is placed on top of the GO nanoscale layer. Figure 4 below shows an AFM image for the GO layer deposited on a silicon wafer by spin-coating. It is evident that the GO coats the wafer quite uniformly, where most of the surface is covered with 1–2 nanosheets forming a continuous percolated layer. Thus, the result of the electrical measurements (which showed that the GO spin-coated layer does not conduct electricity) is not connected to the possible formation of a discontinuous “island-like” GO layer on the surface of the wafer. We note that a small fraction of the surface is not coated with GO. Quantitative AFM bearing analysis of the topographical image indicated that about 80–90 % of the wafer is covered with the nanosheets. We determined the thickness of the single nanosheets from a cross-sectional analysis of the topographical image (Figure 4a) and found that their thickness is about 0.8–1.2 nm, close to the thickness provided by the manufacturer [33]. Root-mean-square roughness of the GO layer is about 0.7 nm. The low value of the roughness additionally confirms the uniform and even coverage of the substrate with the nanosheets. Finally, the phase image (Figure 4b), which is especially sensitive to surface heterogeneity [45], indicates that the GO nanosheets are uniform on the micron/submicron scale. The imaging data also shows the edges of the GO plates, constituting a minute fraction of the GO layer surface. Therefore, as it is well-known, the edges of the GO sheets in our layer have a different chemical composition than the basalt plane of the sheets [42]. In general, we can summarize that the overwhelming majority of the P3HT macromolecules deposited on the GO layer are in contact with the basalt plane of the nanosheets. Only a small fraction of P3HT chains contacts the SiO_2_ surface and the GO edges.

**Figure 5:**
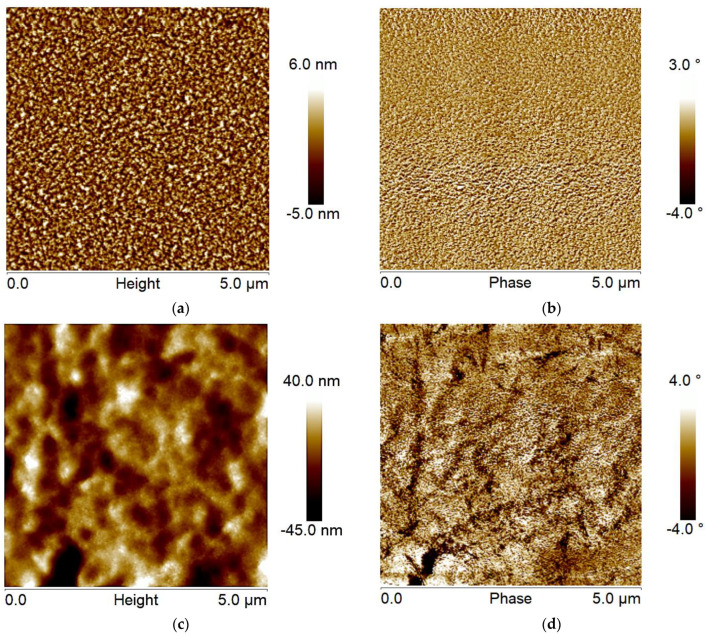
AFM topography (**a**,**c**) and phase (**b**,**d**) images of P3HT layer on SiO_2_ (**a**,**b**) and GO (**c**,**d**) surfaces, where the GO was deposited onto the SiO_2_ layer.

In Figure 5, AFM images show the morphology of the P3HT layer deposited onto a SiO_2_ surface and the P3HT layer deposited onto a SiO_2_ covered with GO layer. The major purpose of the imaging was to examine the P3HT layer in terms of the submicron structure of grain formation and evenness of surface coverage. It is well known that the difference in grain size significantly impacts the movement of charge carriers in the channel of the transistors [46,47,48]. The small grain size leads to the high content of grain boundaries, which results in the formation of trapping sites hindering the movement of charge carriers along the device’s channel. The total mobility of charge carriers in the channel can be divided into two components: mobility in the grain and mobility in the grain boundary [49]. These two mobility components are connected in series, and the resulting effective mobility can be written as [49]:

(8) $$\frac{1}{\mu }=\frac{1}{\mu_{g}}+\frac{1}{\mu_{b}}$$

where $\mu_{g}$ and $\mu_{b}$ are the mobility in the grain and the grain boundary, respectively. According to Equation (8), the mobility in the grain boundary limits the overall mobility of the organic semiconductor. Materials with a distribution of small grain sizes will have significantly more grain boundaries compared to materials containing larger grain sizes. Therefore, the effective mobility of transistors based on active polymers containing small grain sizes will be lower than those containing large grain size polymer layers.

It appears that the surface of the P3HT layer deposited on the SiO_2_ surface has a well-developed grainy structure, as shown in Figure 5a. A cross-sectional and particle analysis of the topographical image reveals that the grain size is on the level of 80–200 nm. RMS roughness of the layer is ~2 nm indicating that the apparent grain height is about 4 nm. Since the thickness of the layer is about 58 nm (which is smaller than the lateral grain size), we suggest that the grains are the major structural element of the P3HT layer in S-OTFTs. Figure 5b shows the phase image of the P3HT layer placed on SiO_2_. At first approximation, the phase image mostly follows topographical features on the surface. However, smaller-scale, “ripple-like” surface corrugations are also observed. The cross-sectional analysis of the phase images indicated that the lateral size of the corrugations is about 30–60 nm (as measured by AFM). We suggest that the corrugations reflect the packing of P3HT macromolecules on the surface into lamellar structures, as reported elsewhere [14,16,50,51,52].

The P3HT layer deposited over the GO layer has entirely different structural features, as shown in the topographical image in Figure 5c. Specifically, much larger grains merging into continuous ribbon-like structures are observed. The grain size (and lateral size of the ribbon) is on the level of 300–600 nm, as determined from the cross-sectional analysis of the images. RMS roughness of the ribbon-like layer is ~12 nm indicating that the height of the surface structures is about 24 nm. As for the P3HT/GO phase image shown in Figure 5d, it mostly follows topographical features on the surface, and, in addition, the “ripple-like” surface corrugations (lamellar structures with 40–60 nm lateral dimensions) are observed as well. We note that for the S-OTFT and M-OTFT, the height of the domains is significantly smaller than the thickness of the P3HT layer. Thus, the surface of the dielectric is covered with the polymer. So, we suggest that the larger grain size of the P3HT channel of the transistors containing the GO interfacial layer is one of the P3HT layer parameters that lead to higher effective mobility than that of the S-OTFTs. 

It was not obvious why there was so dramatic a difference in the morphology of P3HT layers deposited on the SiO_2_ and GO surfaces. One of the possibilities is that the initial stages of P3HT dewetting cause the formation of the grains from the dielectric substrates during the annealing procedure. Thus, the dielectric and P3HT surface energies can be significant factors that affect the morphology of the P3HT layer and, consequently, the device’s performance. Here, the important parameter of interest is the spreading coefficient, which can be expressed as [53]:

(9) $$S_{1-2}=\gamma_{1}-\;\gamma_{2}-\gamma_{1-2}$$

where *γ*_1_ is the surface energy of a substrate, *γ*_2_ is the surface energy of a polymer deposited on the surface, and *γ*_1−2_ is interfacial energy for polymer-substrate contact. From thermodynamical considerations, *S*_1−2_ must be positive for the spontaneous spreading of a polymer liquid over a substrate. If the spreading coefficient is negative, a polymer liquid will bead up (dewet) on the surface. To estimate *S*_1−2_ we determined the surface energy of SiO_2_ and GO layers using the Owens-Wendt model and interfacial energy for P3HT/SiO_2_ and P3HT/GO contacts [54,55]. For P3HT, surface energy and its components available from the scientific literature [56] were used in our calculations. The procedure is described in Supporting Information (SI). We found that *S*_1−2_ values are positive and have very close values of 5.8 mN/m and 5.9 mN/m for SiO_2_ and GO, respectively. Thus, P3HT has no thermodynamic tendency to dewet from either surface.

Since there is no thermodynamic tendency for dewetting of P3HT from GO, we suppose the surface morphology of the GO layer is the decisive factor in the formation of the P3HT layer with significant surface roughness. In fact, from careful observation of the phase image for the P3HT/GO layer (Figure 5d), one can see that the elevations on the surface originate from the “line-like” depressions. These features reflect the presence of the nanosheet edges on the GO layer. Therefore, we suggest that the origin of the relatively rough P3HT/GO layer is the surface topography. Namely, the edges of the GO nanosheets, to a certain extent, guide the spreading of the polymer solution during spin-coating. In fact, it is well established that topographical features on the surface, along with wettability, solution concentration, and spin speed/duration, affect the morphology of the spin-coated polymer films [57].

#### 3.2.2. Assessment of the Interfacial Interactions

It is known that interfacial interaction between a dielectric surface and P3HT can play an important role in the behavior of the organic polymer semiconductor and, therefore, OTFT performance [17,18]. In fact, carrier transport occurs in the conductive channel within a few molecular layers near the dielectric boundary [8,14,15,16,58]. However, it is necessary to point out that the interaction plays a significant role in OTFT fabrication even before the device is in use. Specifically, during the formation of the deposited P3HT layer, the attraction between the polymer and substrate influences the structurization of the layer during the annealing. In the layer deposited in the course of spin-coating, where a solvent is rapidly evaporating, the polymer chains are not in an equilibrium state. The equilibration occurs during the thermal treatment of the layer above the polymer’s glass transition temperature. The interaction between the polymer and substrate can significantly affect the equilibration process. Namely, as the interfacial interaction is high, chain rearrangement can be restricted by surface/polymer affinity. The level of P3HT/substrate thermodynamic interaction can be estimated via interfacial tension, where higher tension values can be related to the lower level of attraction between substrate and polymer [53,59]. We estimated the interfacial tension values (Supporting Information), which are 32.6 mN/m and 40.4 mN/m for P3HT/SiO_2_ and P3HT/GO, respectively. The calculations indicate that P3HT has a higher affinity to the silicon oxide surface. Thus, it is the first indication that we can associate the smaller size of the grains for the P3HT/SiO_2_ layer with difficulty forming the larger grains because of decreased diffusivity of the macromolecules physically connected to the substrate. 

To put this argument in a molecular-level perspective, we estimated the size of the P3HT macromolecules (end-to-end distance, *R*) and compared the obtained value with the thickness of the polymer layer [60]:

(10) $$R={\sqrt{C}}_{\infty \;}\sqrt{n}\;l_{0}$$

where *C*_∞_ is the characteristic ratio (*C*_∞_ = 12), *n* is the number of monomeric units in a P3HT chain (*n* = 361 for our polymer having molecular weight of ~60,000 g/mol), and *l*_0_ is length of one 3HT monomer (*l*_0_ = 0.39 nm). We calculated *R* to be 26 nm. Thus, the P3HT layer used in the devices here (thickness ~58 nm) has dimensions on the level of 2 macromolecular layers. Thus, about half of the polymer chains constituting the layer are in physical contact with the substrate surface, and the movement of their segments is restricted [61,62].

It is necessary to point out that P3HT/substrate interaction at the molecular level between SiO_2_ and GO can be quite significant. It has been previously shown that Si-OH functionalities located on the SiO_2_ surface can induce a certain P3HT chain orientation, which tends to be perpendicular to the insulator substrate (edge-on structure) [14,15,16]. This orientation is caused by the repulsive forces between π-electron clouds of the thienyl backbone and unshared electron pairs of -OH functionality and is shown to have higher carrier mobility than face-on orientation, where thienyl rings are orienting parallel to the surface. On the other hand, the absorbed water molecules and irregularly located Si-OH groups on the SiO_2_ surface can lead to interface trapping and hysteresis [63]. Conversely, GO nanosheets have a distinctive mosaic structure containing disordered oxygen-containing and ordered graphitic regions [42]. The oxygen-containing species are a mixture of hydroxyl, carbonyl, carboxyl, and epoxy functionalities. The typical content of graphitic sp^2^ carbon regions is about 40%. The regions have a size of mainly 1–10 square nm [64,65], which is much smaller than the size of a P3HT macromolecule with an end-to-end distance of ~26 nm and projection on the surface of about 530 nm^2^. Therefore, each P3HT macromolecule is in contact with various surface functionalities and cannot adopt a certain preferred conformation and structurization as shown for the SiO_2_ surface and thus can have (from a molecular level consideration) a higher level of diffusivity during the annealing.

To this end, to assess the difference in mobility of polymer chains in contact with SiO_2_ and GO on a qualitative level, we prepared three model materials and investigated their thermal behavior with DSC between −100 °C and 150 °C. Indeed, it has been demonstrated elsewhere that the glass transition, T_g_, of polymers, which is associated with the coordinated movement of the polymer chain segments, is significantly affected by the presence of a solid boundary [61,62,66,67]. Specifically, when there is a certain level of attraction between polymer chains and the surface, T_g_ for the bonded chain segments is higher than that for the macromolecules not located near the surface. The transition for the bonded chain segments often cannot be detected in an experiment because of the severe restriction in their mobility [62,66]. 

The model materials were mixtures of SiO_2_ submicron particles and GO nanosheets with P3HT as follows: P3HT/SiO_2_ (1:1 weight ratio), P3HT/GO (1:1 weight ratio), and P3HT/GO/SiO_2_ (1:1:1 weight ratio). The latter sample was prepared by premixing SiO_2_ and GO in water, drying the mixture, and its redispersion in chloroform (not a good dispersive media for polar SiO_2_ particles). Then, the dispersion in chloroform was mixed with a P3HT solution and dried. Since the surface of GO nanosheets is orders of magnitude higher than that of the particles, we envisioned that the sample represents a situation where the SiO_2_ surface is shielded with GO. The materials were annealed at 110 °C prior to the DSC measurements to replicate the thermal conditions used to prepare OTFTs. We added a large amount of SiO_2_ and GO fillers to the polymer matrix to maximize the extent of contact between the macromolecules and the surface. For instance, straightforward geometrical calculations [68] using the size of SiO_2_ particles (diameter 500 nm) and densities of P3HT (1.1 g/cm^3^) [69] and SiO_2_ (2 g/cm^3^) [61] show that the distance between the particles in the P3HT/SiO_2_ mixture is on the level of 100 nm. So about 40–60% of the polymer chains are in contact with the surface. It is close to the situation estimated for the P3HT layer in the S-OTFTs. Certainly, for GO, having a significantly higher surface-to-volume ratio, practically all polymer chains are in the vicinity of the nanosheets in our model materials. Thus, a large fraction of macromolecular segments physically contacts the GO surface.

**Figure 6:**
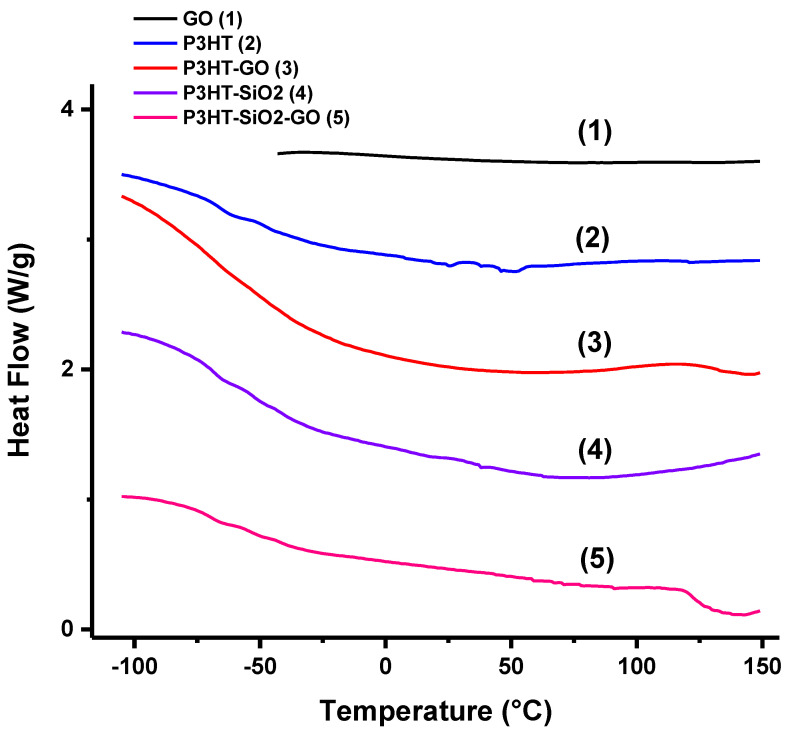
DSC curves for the model materials.

The DSC data for the model materials, as well as for pure P3HT and GO nanosheets (annealed under the same conditions), is presented in Figure 6. We did not conduct measurements for pure SiO_2_ particles since DSC data for them is available from the scientific literature [70]. First of all, we note that GO does not have any thermal transitions in the temperature region studied. For the pure P3HT, a typically found thermal behavior with a quite broad glass transition region is observed. The T_g_ reported in the scientific literature for P3HT varies from −113 °C to 106 °C depending on chemical structure, synthetic procedure, and sample preparation method [71,72,73,74,75]. For our pure P3HT sample, glass transition occurs between −90 °C and −14 °C. Similar thermal behavior of the polymer in the T_g_ region is found for all model samples studied here. Therefore, the diffusion of a significant number of the macromolecular segments in the P3HT matrix is not restricted by the presence of the SiO_2_ and GO solid boundary. When P3HT is mixed with SiO_2_ particles, there is an additional broad and shallow endothermic peak at higher temperatures. It was reported elsewhere that SiO_2_ particles between 40 °C and 180 °C can demonstrate an endothermic peak originating from the volatilization of water on the surface of particles [70]. Similar behavior is seen in our DSC scan for the P3HT/SiO_2_ mixture. For the P3HT/GO samples, we note an additional T_g_-like second-order transition at about 120 °C. The transition is not present in pure P3HT and P3HT/SiO_2_ samples. We associate this transition with the onset of movement of macromolecule segments that are in contact with the GO surface. The same transition was found for the P3HT/GO/SiO_2_ mixture. The transition is even more pronounced for this mixture since the concentration of P3HT is lower in this case, and more chain segments are in contact with the GO surface. Based on the DSC measurements we can qualitatively conclude that P3HT chain segments are more mobile when in contact with GO than when they are in contact with SiO_2_. As mentioned above, we associate this increased mobility with formation of larger P3HT grains in M-OTFTs and therefore, better electrical performance of M-OTFT devices.

## 4. Conclusions

It is demonstrated that the deposition of a monolayer of GO nanosheets on the surfaces of the SiO_2_ dielectric and Au electrodes in P3HT-based OTFTs significantly improves device performance. To our knowledge, this is the first report where unmodified virgin GO is used for this purpose. Specifically, the drain current and the field-effect mobility of OTFTs were considerably increased by modification of the interfaces with GO nanoscale layers. We associate the obtained enhancement with the previously reported [24] decrease in contact resistance for GO-covered electrodes and the particular structure of the P3HT layer on the dielectric surface. Namely, we found a specific morphology of the organic semiconductor P3HT layer, where larger interconnecting polymer grains are formed on the surface of the GO layer deposited on SiO_2_. From a thermodynamic and molecular standpoint, it is suggested that this specific morphology is formed due to the increased mobility of the macromolecular segments in the vicinity of the solid boundary. This increased segment mobility was confirmed via DSC measurements.

## Data Availability

The data that support the reported results are presented in the manuscript and Supporting Information using text, figures, and tables.

## Supplementary Materials

The Supporting Information is available via the internet: Calculation of surface and interfacial energies. The following supporting information can be downloaded at: https://www.mdpi.com/article/10.3390/polym14235061/s1. Table S1: Surface free energy of the dispersive and polar component of hexadecane and water; Table S2: Surface free energy of SiO_2_, GO, and P3HT; Table S3: Interfacial energy of P3HT/SiO_2_ and P3HT/GO. References [54,55,56,76,77,78] are cited in the supplementary materials.
