# Supplementary material for: Poly(3-hexylthiophene)-Based Organic Thin-Film Transistors with Virgin Graphene Oxide as an Interfacial Layer
Source: Polymers (Basel). 2022 Nov 22;14(23):5061. doi: 10.3390/polym14235061 (PMC9736656; doi:10.3390/polym14235061)
Supplement: Supplementary file 1 [file polymers-14-05061-s001.zip › polymers-1956615-Supporting Information.pdf]

SUPPORTING INFORMATION

Poly(3-hexylthiophene)-based Organic Thin-Film Transistors with Virgin Graphene Oxide as an Interfacial Layer

Eyob N. Tarekegn [1], Mastooreh Seyedi [2], Igor Luzinov [2,*] and William R. Harrell [1,*]

[1] Holcombe Department of Electrical and Computer Engineering, Clemson University, Clemson, SC 29634, USA
[2] Department of Materials Science and Engineering, Clemson University, Clemson, SC 29634, USA
* Correspondence: luzinov@clemson.edu (I.L.); wharrel@clemson.edu (W.R.H.)

Calculation of Surface and Interfacial Energies

In the Owens-Wendt model, the surface energy is divided into two components: a polar component and a dispersive component of non-polar interaction. Therefore, the surface energy is given by:[1-2]

$$\gamma_S = \gamma_S^d + \gamma_S^P \qquad \textbf{(S1)}$$

where γ_S is the surface free energy of a solid; γ_S^d and γ_S^P are the dispersive and polar components of the surface free energy of a solid, respectively. The dispersive and polar components of the surface free energy of a solid can be determined using the following equation:[3-4]

$$\gamma_L(1 + cos\theta) = 2\sqrt{\gamma_S^d \gamma_L^d} + 2\sqrt{\gamma_S^P \gamma_L^P} \qquad \textbf{(S2)}$$

where γ_L is the surface free energy of an immersion liquid, γ_L^d and γ_L^P are the surface free energy of the dispersive and polar components of an immersion liquid, respectively, and θ is the contact angle of a liquid on the solid surface. In Equation **S2**, since the values for γ_L^d, γ_L^P and γ_L (where $\gamma_L = \gamma_L^d + \gamma_L^P$) are known[5], and θ can be measured, we have only two unknowns, γ_S^d

and γ_S^P; therefore, we need two contact angles from two different liquids to solve for the two unknowns. **Eq. S2** can be re-written for the two liquids as follows:

$$\gamma_{L1}(1 + cos\theta 1) = 2\sqrt{\gamma_S^d \gamma_{L1}^d} + 2\sqrt{\gamma_S^P \gamma_{L1}^P} \quad \textbf{(S3)}$$

$$\gamma_{L2}(1 + cos\theta 2) = 2\sqrt{\gamma_S^d \gamma_{L2}^d} + 2\sqrt{\gamma_S^P \gamma_{L2}^P} \quad \textbf{(S4)}$$

The two liquids used to measure the contact angles on the SiO_2 and GO surfaces are hexadecane ($C_{16}H_{34}$) and water. Hexadecane was chosen for its non-polar nature, and water was chosen for its polar nature. The standard deviation for the contact angle measurements was $\pm$ 2 degrees. The surface free energy of the dispersive and polar components of hexadecane and water are shown in **Table S1**.

Table S1: Surface free energy of the dispersive and polar component of hexadecane and water (taken from Ref. 5)

	**Water**	**Hexadecane**
γ_L^d (mN/m)	21.8	26.35
γ_L^P (mN/m)	51	0
γ_L (mN/m)	72.8	26.35

The contact angles of water on the SiO_2 and GO surfaces are 28˚ and < 5˚, respectively. The contact angles of hexadecane on SiO_2 and GO surfaces are < 5˚. The dispersive and polar components of the surface free energy of SiO_2 and GO are determined using **Eqs. S3** and **S4**, and the results are shown in **Table S2**. The surface free energy (γ_S) of SiO_2 and GO are determined

using **Eq. S1**, and the results are also shown in **Table S2**. Surface energy and its components for P3HT (available from the scientific literature)[6] are also presented in **Table S2**.

Table S2: Surface free energy of SiO_2, GO, and P3HT.

	**SiO_2**	**GO**	**P3HT**
γ_S^d (mN/m)	26.35	26.4	24.6
γ_S^p (mN/m)	38.9	46.8	2.3
γ_S (mN/m)	65.25	73.2	26.9

After obtaining the surface energies of all the materials (**Table S2**), the interfacial energy between two materials can be calculated using the following equation**:**[1]

$$\gamma_{1-2} = \gamma_1 + \gamma_2 - \frac{4\gamma_1^d\gamma_2^d}{\gamma_1^d+\gamma_2^d} - \frac{4\gamma_1^p\gamma_2^p}{\gamma_1^p+\gamma_2^p} \quad \text{(S5)}$$

The interfacial energy of P3HT/SiO_2 and P3HT/GO interfaces are determined using **Eq. S5**, and the results are shown in **Table S3**.

Table S3: Interfacial energy of P3HT/SiO_2 and P3HT/GO

	P3HT/SiO_2	P3HT/GO
γ_{1-2}(mN/m)	32.58	40.39

References

1. Wu, S., Calculation of interfacial tension in polymer systems. *Journal of Polymer Science Part C: Polymer Symposia* **1971,** *34* (1), 19-30.

2. Luzinov, I.; Xi, K.; Pagnoulle, C.; Huynh-Ba, G.; Jérôme, R., Composition Effect on the Core–Shell Morphology and Mechanical Properties of Ternary Polystyrene/Styrene–Butadiene Rubber/Polyethylene Blends. *Polymer* **1999,** *40* (10), 2511-2520.
3. Owens, D. K.; Wendt, R. C., Estimation of the surface free energy of polymers. *J. Appl. Polym. Sci.* **1969,** *13* (8), 1741-1747.
4. Żenkiewicz, M., Methods for the calculation of surface free energy of solids. *Journal of achievements in materials and manufacturing engineering* **2007,** *24*, 137-145.
5. Janczuk, B.; Wojcik, W.; Zdziennicka, A.; Bruque, J. M., Components of the surface free energy of low rank coals in the presence of n-alkanes. *Powder Technology* **1996,** *86* (3), 229- 238.
6. Wang, X.; Ederth, T.; Inganäs, O., In Situ Wilhelmy Balance Surface Energy Determination of Poly(3-hexylthiophene) and Poly(3,4-ethylenedioxythiophene) during Electrochemical Doping−Dedoping. *Langmuir* **2006,** *22* (22), 9287-9294.
